# Leukoencephalopathia, demyelinating peripheral neuropathy and dural ectasia explained by a not formerly described de novo mutation in the *SAMD9L* gene, ends 27 years of investigations – a case report

**DOI:** 10.1186/s12883-019-1319-1

**Published:** 2019-05-03

**Authors:** Sofia Thunström, Markus Axelsson

**Affiliations:** 1 Department of Clinical Neuroscience and Rehabilitation, Institute of Neuroscience and Physiology, The Sahlgrenska Academy, University of Gothenburg, Gothenburg, Sweden; 2 Department of Clinical Genetics, Sahlgrenska University Hospital, Gothenburg, Sweden; 3 Department of Internal Medicine and Clinical Nutrition, Institute of Medicine, Sahlgrenska Academy, Gothenburg University, Gothenburg, Sweden

**Keywords:** Leukoencephalopathia, Demyelinating peripheral neuropathy, Dural ectasia explained, de novo mutation, The *SAMD9L* gene

## Abstract

**Background:**

Missense mutations in *SAMD9L* gene is associated with ataxia-pancytopenia syndrome (ATXPC), OMIM#159550. Common clinical features in these patients include neurological and hematological symptoms. The phenotype and age of onset is variable.

**Case presentation:**

In this case report whole exome sequencing (WES) revealed a not previously reported de novo variant c.2686 T > G, p.(Phe896Val) in *SAMD9L* in a patient with widespread findings of slow developing pathology in the peripheral and central nervous system. The clinical picture was dominated by neurological symptoms, unlike previously described cases, and in addition dural ectasias and multiple cysts in the brain was observed using magnetic resonance imaging.

**Conclusions:**

This case underscores the effect of variable expressivity, i.e. different mutations in the same gene can cause different phenotypes.

## Background

Mutations in the *SAMD9L* gene, located in chromosome 7, are primarily associated with the ataxia-pancytopenia syndrome (ATXPC) [1], myeloplasia and leukemia syndrome with monosomy 7 (MLSM7) [2]. The *SAMD9L* protein is expressed in a variety of human tissues although the exact role remains obscure. The gene seems to have an antiproliferative function and acts as a tumor suppressor in a number of types of carcinomas [3]. In hematopoietic tissue it affects the homotypic endosome fusion, and thereby the degradation of the cytokine receptors [4].

Engagement of the central nervous system is to some degree evident in most carriers of pathogenic *SAMD9L*. Most evident are the cerebellar symptoms of early onset of balance problems and nystagmus followed by mild pyramidal signs [5]. On magnetic resonance imaging cerebellar atrophy is often seen, and in some cases mild white matter hyperintensity can be detected. The neurological symptoms are generally mild and slowly progressive and usually remain moderate lifelong [6–8].

## Case presentation

**Fig. 1:**
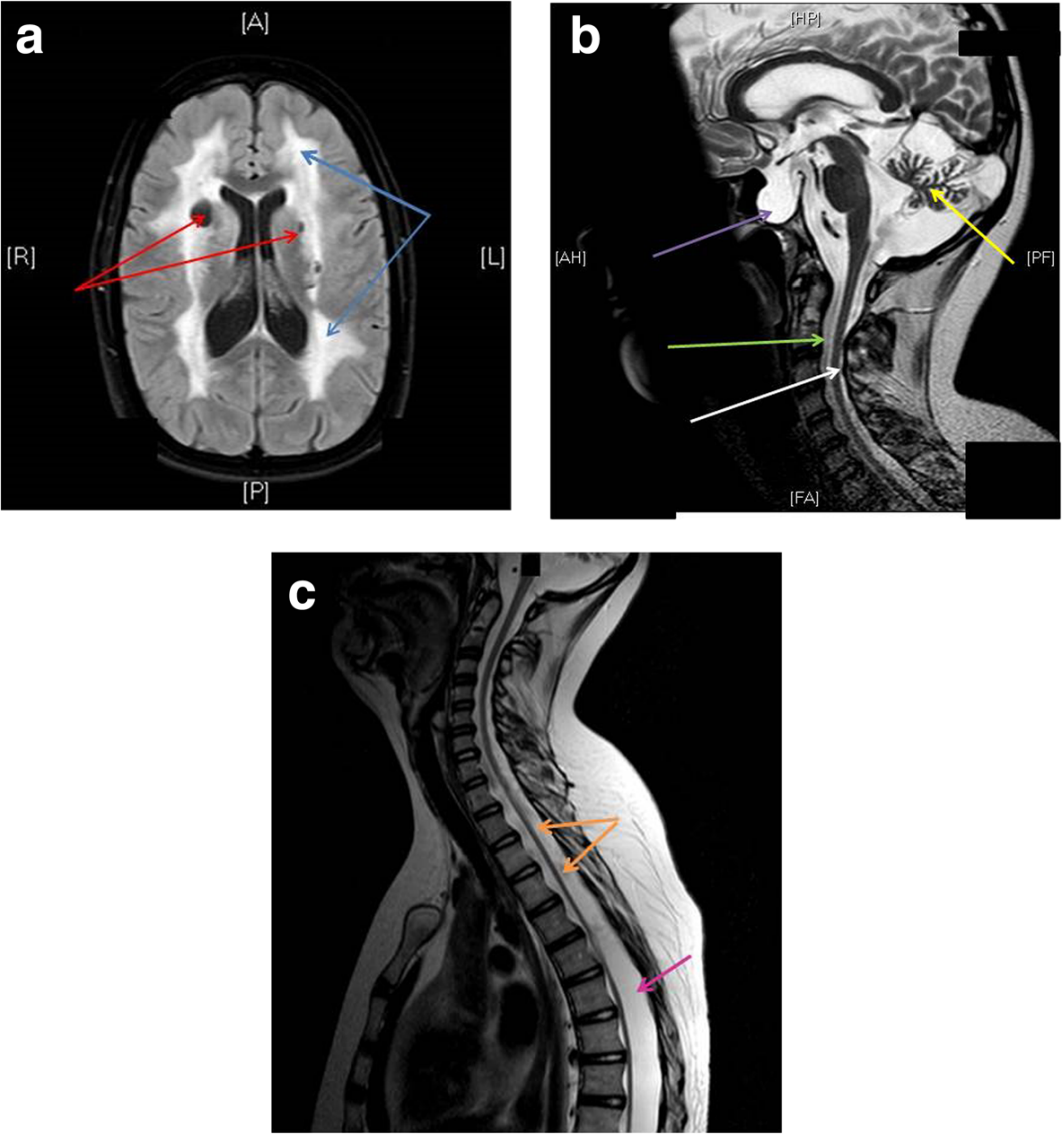
**a** MRI t2 FLAIR (transversal). Blue arrows: Confluent hyperintense signal of unknown origin engaging most of the white matter. Red arrows: Cysts in the white matter with the same signal as CSF. **b** MRI t2 (sagital). Purple arrow: Emty sella tursica partially filled with dura. Hypophysis compressed in dorsal direction. Green arrow: Dural ectasia. White arrow: Medullar atrophy. Yellow arrow: Cerebellar atrophy. **c** MRI t2 (sagittal). Orange arrow: Medullar atrophy. Whole medulla is affected. Pink arrow: Dural ectasia along the whole length of the medulla

The girl was born at a gestational age of 40 weeks and her birth weight was 3530 g. She had feeding difficulties and at three months of age she was hospitalized after subacute developing of paleness and petechiae. A pancytopenia was found with critical low levels of thrombocytes as well as moderate anemia and lymphopenia. She recovered spontaneously, but the cause of the pancytopenia remained unclear. At the age of five it was obvious that she was both slower and weaker than other children of her age, thus a neurological investigation was initiated. She ran with clumsiness and had difficulties standing on heels and toes, and she had weakened tendon reflexes. Nerve conduction studies and electromyography suggested demyelinating neuropathy and hereditary polyneuropathy was suspected. At eight years of age the investigation was reactivated since nystagmus and lively tendon reflexes in patella had developed. Her cognitive function and general development were normal. MRI showed (Fig. 1) abnormal signaling in white matter and disseminated cysts in white and grey matter and a pronounced atrophy of the cerebellum. No medical reason was found that could explain these findings. For the following seven years she was followed at her local hospital, and was considered clinically stable. But at the age of 13 she developed an intra cerebral hemorrhage and an extensive investigation was therefore performed at the university hospital. A new MRI scan of the brain and the medulla were conducted. The brain was assessed as unchanged (except for the hemorrhage). However, the MRI of the medulla revealed long gone dural ectasias involving sella tursica resulting in a mechanical pressure on the hypophysis. EEG was normal and no signs of pathology were observed in the collected cerebrospinal fluid (CSF) samples except for slight increased levels of neurofilament light chain. In addition, generalized joint laxity was observed and this generated the hypothesis that she could suffer from a soft tissue disease or a mitochondrial disease. Muscle biopsy and genetic testing regarding Charcot-Marie-Tooth disease type 1A (*CMT1A*), Friedrichs ataxia (trinucleotide repeat in *FXN*), Loeys-Dietz syndrome (*TGFBR1, TGFBR2*) and Ehler Danlos syndrome type IV (*COL3A1*) were all normal as well as the karyotype of lymphocytes (46,XX). She did fully recover from the stroke associated symptoms, but she had problems walking without support (walked 10 m without support and used wheelchair otherwise), a finding not attributed to her cerebral bleeding. Her gait disturbance was caused by the paresis of central origin in her legs. If it could be explained by the pathology in the brain or the spinal cord or both could not be determined. Although the cerebellum was seemed atrophied on MRI the only obvious clinical features from that area was nystagmus. No explanation was found for her widespread symptoms. During the remainder of her youth she continued with regular follow-ups at her local hospital. At 18 years of age she was referred to the department of neurology at the university hospital. No further focal neurological deficits were developed during the following years but she is considered mentally fatigued. At the age of 27 whole exome sequencing (WES) was performed.

## Sequencing results

Patient was heterozygous for a de novo variant in SAMD9L; NM_152703.4:c.2686 T > G, p.(Phe896Val), no signs of mosaicism in the WES or Sanger analyses.. The variant has previously not been reported to our knowledge. It is a missense mutation and generates an amino acid change, phenylalanine to valine, in a highly conserved region. In silico tools (SIFT, Mutation Taster, Align GVGD and PolyPhen2) predict the variant as deleterious. There was one more variant detected in *SAMD9L*, inherited from the patients healthy father, NM_152703.4:c.1565C > T, p.(Ala522Val) with an allele frequency of 1,7% in SweGen database and 1,4% in Exome Aggregation Consortium (ExAC) database, We could not exclude cis with c.2686 T > G by Integrative Genomics Viewer (IGV). The variant was interpreted as not disease causing due to its allele frequency and inheritance from an healthy individual. Analysis of WES data of chromosome 7q showed no signs of acquired UPD(7q).

## Whole exome sequencing and data filtering

Extraction of DNA was performed from whole blood using QIA Symphony DSP DNA Mini Kit (Qiagen, Hilden, Germany), according to the manufacturer’s instructions and was subjected to WES. DNA library preparation and exome capturing was preformed using the Agilent Sureselect Clinical Research Exome v2. Sequencing was undertaken with paired-end 150 base pair reads using Illumina NextSeq500.

Sequence reads were aligned to the reference genome (hg19) using CLC biomedical workbench and variant calling was also performed with CLC biomedical workbench. Filtering of called variants was performed in several steps using Bench Lab NGS. We analyzed known disease-causing genes within the OMIM database. Further filtering was done to predict pathogenic variants (using SIFT, Mutation Taster, Align GVGD and PolyPhen2) and the level of conservation. How common the variants are in the population (using ExAC, Exome Aggregation Consortium project, 1000 Genome; NHLBI Exome Sequencing Project (ESP)) were investigated to reduce the number of variants.

Confirmation of variants and analysis of parental samples was undertaken by Sanger sequencing according to standard procedures of the manufacturer (Thermo Fisher Scientific) Genomic DNA from the patient and the parents were analyzed.

## Discussion and conclusions

Mutations in the SAMD9L are previously described as associated with the myelocerebellar disorder ATXPC (OMIM#159550). All reported mutations have so far been missense mutations, like our case. To this date five families have been described and some sporadic de novo cases [9–11]. Almost all of the known cases have predominantly hematological symptoms, unlike our patient where the major findings were from the nervous system. Her transient pancytopenia and progressive cerebellar symptoms are previously described in other patients with defects in the same gene. However this patient may extend the phenotype as the cysts in the CNS, the dural ectasias and cerebral hemorrhage are not previously described. One could speculate if these symptoms represent an additional rare disease not found by the WES or to some extent could be secondary to a hematological defect. This case underscores the effect of variable expressivity, i.e. different mutations in the same gene can cause different phenotypes. In this case the clinical evaluation was complex. Large resources have been put into the diagnostics work-up at three different periods over 27 years, initiated by new clinical symptoms. Even so, all efforts stranded in lack of diagnostic tools and previously described combination of progressive symptoms and clinical findings. The whole exome sequencing finally resolved the long lasting riddle of her disease. Hence, we recommend a wider use of whole exome sequencing in cases of hard interpreted combinations of neurological symptoms in an earlier stage.

## Abbreviations

ATXPC: Ataxia-pancytopenia syndrome
CMT1A: Charcot Marie Tooth type 1A
CSF: Cerebrospinal fluid
EEG: Electro Encephalogram
MRI: Magnetic resonance Imaging
WES: Whole exome sequencing

## References

[1] Shannon KM, Turhan AG, Chang SS (1989). Familial bone marrow monosomy 7. Evidence that the predisposing locus is not on the long arm of chromosome 7. J Clin Invest.

[2] Li FP, Potter NU, Buchanan GR, Vawter G, Whang-Peng J, Rosen RB (1978). A family with acute leukemia, hypoplastic anemia and cerebellar ataxia: association with bone marrow C-monosomy. Am J Med.

[3] Phowthongkum PCD, Raskind WH, Bird T (1993). SANDL9-related ataxia-pancytopeniasyndrome.

[4] Nagamachi A, Matsui H, Asou H (2013). Haploinsufficiency of SAMD9L, an endosome fusion facilitator, causes myeloid malignancies in mice mimicking human diseases with monosomy 7. Cancer Cell.

[5] Gorcenco S, Komulainen-Ebrahim J, Nordborg K (2017). Ataxia-pancytopenia syndrome with SAMD9L mutations. Neurol Genet.

[6] Schmitz-Hubsch T, Coudert M, Giunti P (2010). Self-rated health status in spinocerebellar ataxia--results from a European multicenter study. Mov Disord.

[7] Wictorin K, Bradvik B, Nilsson K (2014). Autosomal dominant cerebellar ataxia with slow ocular saccades, neuropathy and orthostatism: a novel entity?. Parkinsonism Relat Disord.

[8] Ygland E, Taroni F, Gellera C (2014). Atypical Friedreich ataxia in patients with FXN p.R165P point mutation or comorbid hemochromatosis. Parkinsonism Relat Disord.

[9] Cheah JJC, Brown AL, Schreiber AW, Feng J, Babic M, Moore S, Young CC, Fine M, Phillips K, Guandalini M, Wilson P, Poplawski N, Hahn CN, Scott HS. A novel germline SAMD9L mutation in a family with ataxia-pancytopenia syndrome and pediatric acute lymphoblastic leukemia. Haematologica; 2019. 10.3324/haematol.2018.207316. Epub ahead of print. 10.3324/haematol.2018.207316 PMC6601105 30923096

[10] Chen DH, Below JE, Shimamura A (2016). Ataxia-pancytopenia syndrome is caused by missense mutations in SAMD9L. Am J Hum Genet.

[11] Tesi B, Davidsson J, Voss M (2017). Gain-of-function SAMD9L mutations cause a syndrome of cytopenia, immunodeficiency, MDS, and neurological symptoms. Blood..

